# Pennock and Moore on the Action of the Heart

**Published:** 1840-01

**Authors:** 


Art. VI.—*Report of Experiments on the Action of the Heart.* By C. W. Pennock, M.D., &c. and E. M. Moore, M.D. &c.

The experiments detailed in this Report, appear to have been made with care, and under circumstances favorable to accuracy. The conclusions from them coincide very nearly with those of the British physiologists—the correctness of whose results, when compared with those of the French, the authors of the Report believe, is to be attributed to the use of large animals. The following are some of the conclusions:

"1st. The impulse is synchronous with, and caused by, the ventricular contraction,—and when felt externally, arises from the striking of the apex of the heart against the thorax.

"2d. The expulsion of the blood from the ventricles is effected by an approximation of the sides of the heart only, and not by a contraction of the apex towards the base; during the systole the heart performs a spiral movement, and becomes elongated. (Experiments 6th, 10th, and 11th.)

"3d. The ventricle contracts and the auricle dilates at the same time, occupying about one-half of the whole time required for contraction, diastole, and repose. Immediately at the termination of the systole of the ventricle, its diastole succeeds, occupying about one-fourth of the whole time, synchronous with which the auricle diminishes, by emptying a portion of its blood in the ventricle, unaccompanied with muscular contraction. The remaining fourth is devoted to the repose of the ventricles, near the termination of which the auricle contracts actively, with a short, quick motion, thus distending the ventricles with an additional quantity of blood: this motion is propagated immediately to the ventricles, and their systole takes place, rendering their contractions almost continuous. (Experiments 15 and 16.)

"4th. From the termination of their diastole to the commencement of their systole, the ventricles are in a state of perfect repose, their cavities remaining full, but not distended, while those of the auricles are partially so, during the whole time.

"5th. The sounds are produced by the motions of the heart or its contents, and not by striking against the thorax, as proved in all the experiments; being much louder when the stethoscope was applied directly to the heart, than when to the chest, or with the lungs interposed.

"6th. The sounds are more distinct when the muscle is thin, and contracts quickly. Hence, the clear, flapping character of the first sound over the right ventricle, as compared with the left.

"7th. The first sound, the impulse, and the ventricular systole, are synchronous. This sound may be a combination of that caused by the contraction of the auricles, the flapping of the auriculo-ventricular valves, the rush of blood from the ventricles, and the sound of muscular contraction. From experiments 3d, 4th, 6th, and 10th, when the heart was removed from the body, the ventricles cut open and emptied of their contents, the auriculo-ventricular valves elevated, and a sound, resembling the first, still heard, it may be chiefly attributed to the muscular contraction. That these valves aid but slightly in its production, may also be inferred from experiment 16.

"8th. The second sound is caused exclusively by the closure of the semi-lunar valves from the reaction of the arterial columns of blood upon them, in its tendency to regurgitate through the aortic and pulmonary orifices. This is proved by the greater intensity of this sound over the aorta than elsewhere, the blood having a strong tendency to return through the valvular opening; by the greater feebleness of the sound over the pulmonary artery, which is short, and soon distributes its blood through the lungs, thus producing but slight impulse upon the valves in the attempt to regurgitate; by the disappearance of the sound, when the heart becomes congested and contracts feebly; and, finally, on account of its entire extinction when the valve of the aorta was elevated.

"9th. The second sound is synchronous with the diastole of the ventricle."

